# Supplementary material for: Clinician-recalled quoted speech in electronic health records and risk of suicide attempt: a case–crossover study
Source: BMJ Open. 2020 Apr 22;10(4):e036186. doi: 10.1136/bmjopen-2019-036186 (PMC7204853; doi:10.1136/bmjopen-2019-036186)
Supplement: Author's manuscript [file bmjopen-2019-036186.draft_revisions.pdf]

BMJ Open is committed to open peer review. As part of this commitment we make the peer review history of every article we publish publicly available.

When an article is published we post the peer reviewers' comments and the authors' responses online. We also post the versions of the paper that were used during peer review. These are the versions that the peer review comments apply to.

The versions of the paper that follow are the versions that were submitted during the peer review process. They are not the versions of record or the final published versions. They should not be cited or distributed as the published version of this manuscript.

BMJ Open is an open access journal and the full, final, typeset and author-corrected version of record of the manuscript is available on our site with no access controls, subscription charges or pay-per-view fees (http://bmjopen.bmj.com).

If you have any questions on BMJ Open's open peer review process please email info.bmjopen@bmj.com

BMJ Open

Clinicians' Quoted Speech in Electronic Health Records and Risk of Suicide Attempt: A Case-Crossover Study

Journal:	*BMJ Open*
Manuscript ID	bmjopen-2019-036186
Article Type:	Original research
Date Submitted by the Author:	04-Dec-2019
Complete List of Authors:	Jayasinghe, Lasantha; IoPPN Department of Psychological Medicine,<br>Bittar, André; IoPPN Department of Psychological Medicine<br>Dutta, Rina; Kings College London, Institute of Psychiatry, Psychology and Neuroscience; South London and Maudsley NHS Foundation Trust<br>Stewart, Robert; King's College London, Institute of Psychiatry; South London and Maudsley NHS Foundation Trust
Keywords:	PSYCHIATRY, Suicide & self-harm < PSYCHIATRY, Health informatics < BIOTECHNOLOGY & BIOINFORMATICS

SCHOLARONE™
Manuscripts

Clinicians' Quoted Speech in Electronic Health Records and Risk of Suicide Attempt: A Case-Crossover Study

Lasantha Jayasinghe[1], André Bittar[1], Rina Dutta[*,1,2], Robert Stewart[*,1,2]
[1] *Institute of Psychiatry, Psychology and Neuroscience, King's College London, London, UK,*
[2] *South London and Maudsley NHS Foundation Trust, London, UK*

[*]*contributed equally to this work*

Correspondence to: Lasantha Jayasinghe. lasantha.jayasinghe@kcl.ac.uk

Other Author emails:
André Bittar: andre.bittar@kcl.ac.uk
Rina Dutta: rina.dutta@kcl.ac.uk
Robert Stewart: robert.stewart@kcl.ac.uk

Keywords: electronic health records, suicide attempt, mental health, case crossover design, natural language processing

Word count: 2526

For peer review only - http://bmjopen.bmj.com/site/about/guidelines.xhtml

ABSTRACT

Objective: Clinician narrative style in electronic health records (EHRs) has rarely been investigated. Clinicians sometimes record brief quotations of a patient's actual words, and might be more likely to do so when higher risk is perceived. Therefore, this study investigated whether the frequency of quoted phrases in an EHR was higher in time periods closer to a suicide attempt.

Design: A case-crossover design was applied to a set of individuals from a large mental health records database. A natural language processing tool was developed using regular expression matching to identify text occurring within quotation marks in the EHR.

Setting: Electronic records from Clinical Record Interactive Search (CRIS) data resource, a large mental healthcare provider serving a geographic catchment of 1.3 million residents in four boroughs of South London were linked with the Hospital Episode Statistics (HES) database

Participants: 1,503 individuals were identified as having a suicide attempt related hospital admissions from 1st April 2006 to 31st March 2017 with at least one document in both the case period (1 to 30 days prior to admission) and the control period (61 to 90 days prior to admission).

Outcome measures: The number of quoted phrases in the control as compared to the case period.

Results: Both attended and non-attended clinical appointments were independently higher in the case compared to control period, while there was no difference in mental healthcare hospitalisation. In addition, there was no difference in the levels of quoted text between the comparison time periods.

Conclusions: This study successfully developed an algorithm to identify quoted speech in text fields from routine mental healthcare records. Contrary to the hypothesis, no association between this exposure and proximity to a suicide attempt was found; however, further evaluation is warranted on the way in which clinician-perceived risk might be feasibly characterised from clinical text.

For peer review only - http://bmjopen.bmj.com/site/about/guidelines.xhtml

ARTICLE SUMMARY

Strengths and limitations of this study

- A larger sample size (1,503 patients) than previous related studies.
- The case-crossover design eliminates between-patient differences as potential confounding factors, making the results more robust.
- Time-varying factors that could potentially cause confounding were included as covariates in the regression.
- 90% of the 14,960 patients making a suicide attempt in the case period could not be analysed, due to no documentation in the control period, although analysis appears representative.

For peer review only

For peer review only - http://bmjopen.bmj.com/site/about/guidelines.xhtml

INTRODUCTION

Around 800,000 deaths a year internationally are estimated to be the result of suicide (1). In the UK, 5,821 deaths were attributed to suicide in 2017 (2). Given that predictions of suicide risk have not improved significantly in the last 50 years, new data-driven methods would be welcomed, and a recent meta-analysis concluded a shift away from risk factors to risk algorithms (3,4).

Electronic Health Record (EHR) data provide important potential opportunities in this respect, and for patients receiving mental health services they hold a rich source of longitudinal data leading up to recorded suicide attempts. There are also benefits over traditional assessment methods such as questionnaires, in which respondents may not answer accurately due to perceived stigma (5–7) or limited recall (5). Commonly, predictive studies have sought to understand suicide risk in terms of the structured data within EHRs, such as diagnostic codes (8,9). However, recent studies have begun to examine the value of including unstructured EHR data for predicting suicide risk. For example, text-mined EHR data were found to produce more accurate models of suicidal behaviour in a sample of Gulf War veterans (10), and important information such as recorded suicidal ideation and past attempts have been successfully identified in mental healthcare text using natural language processing (11).

For peer review only

The tendency has been to view the EHR as simply a factual summary of a patient's experience and symptoms. However, it is important to bear in mind that the EHR can also be viewed as a narrative account written from the perspective of the clinician and other healthcare professionals (12). Clinician reporting in the EHR varies widely and the nature/style of the reporting may provide additional information beyond what is actually recorded (13). A frequent comment in EHRs is a generic phrase such as 'No evidence of suicidal ideation', yet this raises the question of whether the clinician actually asked about suicide or just observed for signs indicating suicidality. A video analysis of out-patient visits in one study found that most questions about suicidal ideation were closed

For peer review only - http://bmjopen.bmj.com/site/about/guidelines.xhtml

yes/no questions, and three-quarters of the questions were negatively phrased, inviting patients to confirm they were not feeling suicidal (14).

Trainee psychiatrists are often taught to use brief verbatim patient quotes to record a patient's actual words in order to provide better evidence for their decision making and also as part of medical defensive practice (15,16). In EHRs this may manifest in direct quotations of statements made by the patient, or by 'referencing' – where a clinician assigns the source of the text to someone other than themselves. Associations of recording style with perceived risk are supported by a higher relative frequency of third-person pronoun use found in groups of veterans who had died from suicide compared to a group of service users who were still alive (17). In that study the use of a group of words related to referencing did not vary significantly between the two groups, but the authors did not report on directly quoted speech in quotation marks. However, another investigation found that quoting 'he/she says' increased in records of clinician-patient interactions that involved communication of bad news between doctor and patient (18).

Clinical recording style has received little investigation in the field of suicidology. To our knowledge, research into the use of quotation marks to record verbatim patient statements in notes has not been conducted to date. We therefore sought to investigate the hypothesis that the frequency of quoted phrases in an EHR would be indicative of higher perceived immediate risk, in that they would be more frequent in time periods closer to a suicide attempt. This was examined in a sample of patients who had been admitted to hospital for a suicide attempt, using a case-crossover design.

For peer review only

METHODS

Study sample

The mental health records used in this study were de-identified copies of those from the South London and Maudsley (SLaM) NHS Foundation Trust, assembled using its Clinical Record Interactive Search (CRIS) platform, which currently accesses mental healthcare records for over 400,000 patients (19). CRIS, as a data resource for secondary analysis, has IRB approval from Oxford Research Ethics Committee C (reference 18/SC/0372). SLaM provides comprehensive, near-monopoly mental healthcare services to a geographic catchment of around 1.3m residents in four boroughs of south London, as well as some national specialist services. CRIS data have been linked to the Hospital Episode Statistics (HES) database, which contains details of all admissions, accident and emergency attendances and outpatient appointments at NHS hospitals in England (20). This CRIS-HES linkage was used to determine suicide-related hospital admissions for individuals between 1st April 2006 and 31st March 2017 inclusive, identifying 14,960 unique patients with at least one suicide attempt, indicated by the presence of any of the following ICD codes: X6*, X7*, X80-4*, Y1*, Y2*, Y30-4*, Y87*, associated with a hospitalisation lasting at least 24 hours (i.e. starting and ending on different dates). From these, a subset of 1,503 patients (10.0%) were identified who had at least one document from mental healthcare in both the case and control period (see below) and who thus provided sufficient data for the analysis. In addition to the text-derived data, demographics including age, gender and ethnicity were extracted to describe the cohort.

Patient involvement

We did not directly incorporate patient and public involvement (PPI) into this particular study but the SLaM BRC Case Register used in the analysis was developed with extensive PPI and is overseen by a committee that includes service-user representatives.

For peer review only - http://bmjopen.bmj.com/site/about/guidelines.xhtml

Case-crossover design

A case-crossover design (21) was used to compare the occurrence of quoted phrases in clinical text between the period just prior to a suicide attempt and a control period, within the same individual. The generic advantage of this design is that individuals serve as their own controls, and therefore between-patient differences are eliminated as potential confounding factors (22). The index date was the hospital admission date for a first suicide attempt. The case period was defined *a priori* as 1 to 30 days prior to the index date and the control period was set at 61 to 90 days prior to this date.

Time-variant factors

Considering other potential differences between the two comparison time periods, the following variables were considered as confounding factors and included as covariates in the regression: i) number of face-to-face outpatient appointments attended, ii) number of appointments made and not attended, iii) number of inpatient bed days in SLaM.

Identification of quoted speech

A natural language processing (NLP) tool was developed using regular expression matching to identify text occurring within quotation marks in the EHR. To avoid mistaking apostrophes used in contractions for the start of quoted phrase, a quote followed by a sequence (‘c’, ‘d’, ‘e’, ‘m’, ‘n’, ‘s’, ‘t’ , ‘ve’, ‘re’, ‘s’, ‘ll’, ‘all’) was treated as an apostrophe not a quote. A similar check was performed for end quotes. Once a quoted phrase was identified, any sub-quotations occurring within that quote were assumed to be part of the larger quotation. The length of quoted phrases was allowed to vary from one word to more than a paragraph; however, a maximum length of 1,500 characters was applied to avoid extracting the entire text where a quote was not properly closed. Phrases that consisted only of emails or URLs were removed using standard regular expression pattern matching and substitution procedures.

For peer review only - http://bmjopen.bmj.com/site/about/guidelines.xhtml

The performance of the algorithm was tested on random samples of both clinical correspondence and case note documents from CRIS (Table 1), with a precision of 0.98, recall of 0.95 and F-Score of 0.96 across the whole test sample. To test the performance in the model sample, 15 random documents identified by the algorithm as containing quotations and 10 without quotations were examined in both the case and control periods. In summary, 49 documents were evaluated, yielding a precision of 0.92, recall of 0.93, F-score of 0.92 and accuracy 0.86. One document was eliminated from the analysis due to incorrect syntax. The majority of inconsistences between the algorithm and annotated quotations were due to grammatical inconsistences such as omitting one of the pair of quotation marks or mismatching two different quotation mark styles. This was not amended in the regular expression matching pattern as it was felt to be more likely to produce false positives than genuine quotes.

Statistical Analysis

Data were cleaned using standard Python (version 3.6.8) libraries. Statistical analyses (paired *t*-tests and conditional logistic regressions) were performed in R (version 3.6.0). Two-tailed paired *t*-tests were carried out to investigate differences in the number of quoted phrases between the control and case periods. Quotations per token were used to normalise for document length. Main analyses used conditional logistic regression to examine the association between the number of quoted phrases in the control and cases periods.

For peer review only - http://bmjopen.bmj.com/site/about/guidelines.xhtml

RESULTS

Cohort Characteristics

The demographics of the study cohort and the wider sample of patients with at least one suicide attempt in the time period of analysis, are described in Table 2. Of the 1,503 individuals in the cohort the majority were female, the mean age at first presentation to SLaM was 34.8 years (*sd* = 16.1 years), with 64.5% aged 40 or under. The majority were of White European background, and the next largest ethnic group was Black. These characteristics of the study cohort were broadly similar to those of the wider sample (Table 2). Individuals in the study cohort had approximately the same number of documents in the control period (*mean* = 14.7, *sd* = 28.5) and case period (*mean* = 15.3, *sd* = 28.9); *p* = 0.381.

Univariate Analyses

Univariate analyses of the individual covariates were carried out to investigate differences between the case and the control period (Table 3). The univariate distributions were non-normal, displaying high peaks and long tails; however, they were compared with paired *t*-tests, which are robust under this distribution (23). Face-to-face outpatient attendances and non-attendances were significantly higher in the case period compared to the control period, but there was no significant difference in the number of inpatient bed days. With regard to the exposure of interest, no difference was found in the number of quoted phrases for the patient group in the case period compared to the control period.

Conditional Logistic Regression

Univariate and multivariate conditional logistic regression results are presented in Table 4. Considering presence or not of quoted text as a binary variable, although an association of borderline significance was present in the unadjusted model (OR 1.17, 95% CI 0.99 - 1.38), this was attenuated substantially after adjustment for other covariates (OR 1.09, 95% CI 0.91 - 1.30) with the

For peer review only - http://bmjopen.bmj.com/site/about/guidelines.xhtml

majority of the attenuation occurring following adjustment for level of face-to-face contact. The full details of the coefficients in the quotations binary model, including face-to-face contacts, appointments not attended and inpatient bed days as covariates are presented in Table 5. Associations between number of quoted text instances and case vs. control periods were close to the null in all models, while both attended and non-attended appointments were independently higher in case compared to control periods.

For peer review only

For peer review only - http://bmjopen.bmj.com/site/about/guidelines.xhtml

DISCUSSION

To our knowledge, this was the first study using a case-crossover design to examine whether changes in the frequency of clinicians' use of quoted text might serve as an indicator of suicide risk in patients. In summary, we found no association between the frequency of quoted phrases between periods close-to and less-close-to the occurrence of an attempted suicide event, although there were differences in other metrics between the time periods, notably increased numbers of both attended and non-attended appointments closer to the event in question.

Previous research has been sparse on mental health risk implications of different reporting styles in clinical records. As mentioned, one previous study had reported no association between referencing text as a construct and suicidality (17); however, we felt that the association of text in quotation marks was worth evaluating as an alternative marker, not only because of the potential strengths of a case-crossover design, but also because of another study's findings that quoted text used in particular clinical circumstances is associated with higher perceived risk (18). One possible difference between that study and ours is that we used quotation marks to identify quoted phrases, rather than attempting to identify the speaker, or exchanges of views between clinician and patient. Thus, extending the definition of quoted text to the identification of relevant pronouns would be a worthwhile avenue for future research.

Additional findings of our study were that increased face-to-face contact and failure to attend appointments were significantly more common in the case compared to control period and thus were markers of higher risk status. These are clinically plausible associations and support the robustness of the case crossover design for this outcome. Patients with increased face-to-face contact in the case period would presumably have a greater number of documents and potentially quotations than in the control period, whilst this would be less likely in those failing to attend appointments in the case period; supporting this, adjusting the association of interest for face-to-

For peer review only - http://bmjopen.bmj.com/site/about/guidelines.xhtml

face contacts resulted in substantial attenuation, whereas adjusting for non-attendances made little difference to coefficients.

The absent association of interest might possibly have arisen because the use of quotations is a relatively invariant factor based on individual clinician linguistic style. This is certainly possible, as the data for this study were drawn from a much larger data source than the previous studies: for example, a narrative analysis of 4 medical interviews (18) or a sample of 63 veterans with the equivalent number of controls (17). Another key strength of this study is that between-patient confounding factors are eliminated through the case-crossover design, which renders a more robust analysis than that attempted previously, and the positive associations with attended and non-attended appointments supports the robustness of the time period comparison. A limitation of our study is that we were only able to analyse a small sample of the 14,960 patients who had made a suicide attempt during the time period of interest, 79% of whom had no prior contact with SLaM (i.e. no documents at all in the case and control periods), although those analysed appeared representative of the source sample on metrics investigated. Consequently, the findings are limited to patients who were already seeking mental health treatment. Previous research shows that in a geographically diverse study of people dying by suicide in the US, in the month prior to death, only 5% of cases received psychiatric treatment, but over 60% had made primary and secondary care visits in the preceding year (24). Potential linkage of information beyond mental healthcare to primary and acute care might prove useful for predictive modelling, not only in the mining of text but also through a wider variety of structured information on the nature and level of service contact.

In conclusion, this study found that there was no difference in the levels of quoted text for individuals at 1 to 30 days versus 61 to 90 days prior to a suicide attempt. However, the successful identification of quoted speech within mental healthcare EHRs may have other applications, and

For peer review only - http://bmjopen.bmj.com/site/about/guidelines.xhtml

there may be fruitful progress to be made in automating the extraction of such text and analysing what the clinician thinks it is important to emphasise from the patient's account.

For peer review only

For peer review only - http://bmjopen.bmj.com/site/about/guidelines.xhtml

REFERENCES

1. WHO: WHO | National suicide prevention strategies: progress, examples and indicators. WHO , 2019.Available from: https://www.who.int/mental_health/suicide-prevention/national_strategies_2019/en/
2. ONS: Suicides in the UK: 2017 registrations [Internet]. Off. Natl. Stat. , 2018.Available from: https://www.ons.gov.uk/peoplepopulationandcommunity/birthsdeathsandmarriages/deaths/bulletins/suicidesintheunitedkingdom/2017registrations
3. Jaroszewski MC, Chang AC, Nock BP: Risk Factors for Suicidal Thoughts and Behaviors: A Meta-Analysis of 50 Years of Research. Psychological Bulletin , 2016.Available from: http://dx.doi.org/10.1037/bul0000084CITATIONAdvanceonlinepublication.http://dx.doi.org/10.1037/bul0000084
4. Velupillai S, Hadlaczky G, Baca-Garcia E, et al.: Risk Assessment Tools and Data-Driven Approaches for Predicting and Preventing Suicidal Behavior. Frontiers in psychiatry 10: 36, 2019.
5. Denneson LM, Basham C, Dickinson KC, et al.: Suicide Risk Assessment and Content of VA Health Care Contacts Before Suicide Completion by Veterans in Oregon. Psychiatric Services 61: 1192–1197, 2010.
6. Bowers L, Banda T, Nijman H: Suicide Inside. The Journal of Nervous and Mental Disease 198: 315–328, 2010.
7. Schulberg HC, Bruce ML, Lee PW, et al.: Preventing suicide in primary care patients: the primary care physician’s role. General Hospital Psychiatry 26: 337–345, 2004.
8. Tran T, Luo W, Phung D, et al.: Risk stratification using data from electronic medical records better predicts suicide risks than clinician assessments. BMC Psychiatry 14: 76, 2014.
9. Walsh CG, Ribeiro JD, Franklin JC: Predicting risk of suicide attempts over time through machine learning. Clinical Psychological Science 5: 457–469, 2017.
10. Ben-Ari A, Hammond K: Text Mining the EMR for Modeling and Predicting Suicidal Behavior

For peer review only - http://bmjopen.bmj.com/site/about/guidelines.xhtml

among US Veterans of the 1991 Persian Gulf War [Internet]; in 2015 48th Hawaii International Conference on System Sciences. IEEE, 2015 [cited 2019 Jun 19]. Available from: http://ieeexplore.ieee.org/document/7070197/

11. Fernandes AC, Dutta R, Velupillai S, et al.: Identifying Suicide Ideation and Suicidal Attempts in a Psychiatric Clinical Research Database using Natural Language Processing. Scientific Reports 8: 7426, 2018.

12. Aaslestad P: The patient as text: the role of the narrator in psychiatric notes, 1890-1990. CRC Press, 2016

13. Hunter KM, Montgomery K: Doctors' stories: The narrative structure of medical knowledge. Princeton University Press, 1993

14. McCabe R, Sterno I, Priebe S, et al.: How do healthcare professionals interview patients to assess suicide risk? BMC Psychiatry 17: 122, 2017.

15. Assessing and managing risk in mental health services - GOV.UK [Internet]Available from: https://www.gov.uk/government/publications/assessing-and-managing-risk-in-mental-health-services

16. Roth, Lorraine S: Writing progress notes: 10 dos and don'ts [Internet]. MDedge Psychiatry. 4(2):63-66, 2005.

17. Leonard Westgate C, Shiner B, Thompson P, et al.: Evaluation of Veterans' Suicide Risk With the Use of Linguistic Detection Methods. Psychiatric Services 66: 1051–1056, 2015.

18. Van DeMieroop D: The quotative 'he/she says' in interpreted doctor–patient interaction. Interpreting 14: 92–117, 2012.

19. Perera G, Broadbent M, Callard F, et al.: Cohort profile of the South London and Maudsley NHS Foundation Trust Biomedical Research Centre (SLaM BRC) Case Register: current status and recent enhancement of an Electronic Mental Health Record-derived data resource. BMJ open 6: e008721, 2016.

20. Hospital Episode Statistics (HES) - NHS Digital [Internet]Available from:

For peer review only - http://bmjopen.bmj.com/site/about/guidelines.xhtml

https://digital.nhs.uk/data-and-information/data-tools-and-services/data-services/hospital-episode-statistics

21. Maclure M: The Case-Crossover Design: A Method for Studying Transient Effects on the Risk of Acute Events. American Journal of Epidemiology 133: 144–153, 1991.

22. Mostofsky E, Coull BA, Mittleman MA: Analysis of Observational Self-matched Data to Examine Acute Triggers of Outcome Events with Abrupt Onset. Epidemiology 29, 2018.Available from:

23. Peacock JL, Kerry SM, Balise RR: Presenting medical statistics from proposal to publication. Oxford University Press, 2017

24. Ahmedani BK, Simon GE, Stewart C, et al.: Health Care Contacts in the Year Before Suicide Death. Journal of General Internal Medicine 29: 870–877, 2014.

For peer review only

For peer review only - http://bmjopen.bmj.com/site/about/guidelines.xhtml

TABLES AND FIGURES

Table 1. Test set performance metrics for random documents selected to contain the quotation mark characters stated.

Minimum document length set to 500 characters, unless otherwise stated.

Quotation mark characters contained	Number of documents	Number of quotations	Precision	Recall	F-Score	Accuracy
<u>**Clinical correspondence**</u>						
“	10	111	0.96	0.95	0.95	0.91
”	10	50	0.98	0.98	0.98	0.96
"	9*	35	1.00	1.00	1.00	1.00
None of: “, ”, "	10	4	1.00	1.00	1.00	1.00
<u>**Case notes**</u>						
“	10	59	0.98	0.92	0.95	0.90

For peer review only

For peer review only - http://bmjopen.bmj.com/site/about/guidelines.xhtml

”	10	53	0.98	0.92	0.95	0.91
"	10	18	1.00	1.00	1.00	1.00
None of: “, ”, "	10	0	-	-	-	-
At least 1 of: ‘, ', “, ”, "	10	10	0.91	1.00	0.95	0.91
All of: ‘, ’, “, ”, "	10	91	0.99	0.98	0.98	0.97
All of: ‘, ’, “, ”, " and document length > 1000 characters	30	264	0.97	0.92	0.95	0.90
Total	129	695	0.98	0.95	0.96	0.92

* One document removed as it was textually corrupt.

For peer review only

For peer review only - http://bmjopen.bmj.com/site/about/guidelines.xhtml

For peer review only

For peer review only - http://bmjopen.bmj.com/site/about/guidelines.xhtml

Table 2. Demographic characteristics of the study sample (*n* = 1503) in comparison to all patients with hospitalised suicide attempt (N= 14,960)

	Study sample		All suicide admissions	
Demographic Variables	***N***	***% Total***	***N***	***% Total***
**Gender**				
Female	932	62.0%	8463	56.6%
Male	571	38.0%	6488	43.4%
Unknown	0	0.0%	9	0.1%
**Ethnicity**				
White European	1083	72.1%	9805	65.5%
Black	274	18.2%	1512	10.1%
Asian	66	4.4%	615	4.1%
Other	66	4.4%	756	5.1%
Unknown	14	0.9%	2272	15.2%
**Age at index hospitalisation (y)**				
< 16	184	12.2%	1612	10.8%
16-20	205	13.6%	2204	14.7%

For peer review only - http://bmjopen.bmj.com/site/about/guidelines.xhtml

21-30	271	18.0%	3567	23.8%
31-40	310	20.6%	2935	19.6%
41-50	280	18.6%	2615	17.5%
51-60	156	10.4%	1195	8.0%
61+	97	6.5%	829	5.5%
Unknown	0	0.0%	3	0.0%

For peer review only - http://bmjopen.bmj.com/site/about/guidelines.xhtml

Table 3. Univariate Analyses - Paired *t*-test results

	Control period		Case period				
**Variable**	**mean**	**sd**	**mean**	**sd**	**Difference**	**95% CI**	***p*-value**
Face-to-face contact	2.47	3.71	2.92	4.14	0.441	0.225, 0.657	<0.001
Appointment not attended	0.38	1.00	0.47	1.13	0.098	0.036, 0.161	0.002
Inpatient bed days	2.29	7.13	2.05	6.69	-0.242	-0.620, 0.134	0.207
Quotations per token	0.16	0.25	0.16	0.23	-0.0004	-0.0158, 0.0149	0.956

For peer review only

For peer review only - http://bmjopen.bmj.com/site/about/guidelines.xhtml

Table 4. Conditional logistic regression models for the association between levels of quoted speech and time period prior to hospitalised suicide attempt

Characteristic	Odds Ratio	95% CI	*p*-value
Total sample (n = 1503)			
**quotations_binary**			
Unadjusted	1.17	0.99, 1.38	0.073
Adjusted for face-to-face contacts	1.08	0.90, 1.28	0.411
Adjusted for dna	1.16	0.98, 1.37	0.087
Adjusted for no. of inpatient bed days	1.18	1.00, 1.40	0.050
Adjusted for all covariates	1.09	0.91, 1.30	0.346
**quotations_per_token**			
Unadjusted	0.99	0.71, 1.38	0.956
Adjusted for face-to-face contacts	0.93	0.67, 1.31	0.693
Adjusted for dna	0.99	0.71, 1.39	0.971

For peer review only - http://bmjopen.bmj.com/site/about/guidelines.xhtml

Adjusted for no. of inpatient bed days	0.99	0.71, 1.39	0.969
Adjusted for all covariates	0.94	0.67, 1.32	0.735

For peer review only

For peer review only - http://bmjopen.bmj.com/site/about/guidelines.xhtml

Table 5. Full conditional logistic regression model for covariates associated with time period prior to hospitalised suicide attempt

Characteristic	Odds Ratio	95% CI	*p*-value
Total sample (n = 1503)			
Quotations (binary)	1.09	0.91, 1.30	0.346
Face-to-face contacts	1.05	1.02, 1.08	0.001
Appointments not attended	1.15	1.04, 1.26	0.004
Inpatient bed days	0.99	0.98, 1.01	0.211

For peer review only - http://bmjopen.bmj.com/site/about/guidelines.xhtml

AUTHOR STATEMENT

Contributors: RD and RS conceived the study design. LJ wrote the paper and analysed the data. LJ also led the development of the quoted speech algorithm with input from AB. RD and RS provided clinical insight on the paper and supervisory guidance. All authors provided critical input for the paper and approved the submission.

Funding: LJ and RD are part-funded by the NIHR Specialist Biomedical Research Centre for Mental Health at the South London and Maudsley NHS Foundation Trust and Institute of Psychiatry, King's College London. RD is also funded by a Clinician Scientist Fellowship (project e-HOST-IT) from the Health Foundation in partnership with the Academy of Medical Sciences, which also funds AB. RS is part-funded by: i) the NIHR Specialist Biomedical Research Centre for Mental Health at the South London and Maudsley NHS Foundation Trust and Institute of Psychiatry, King's College London; ii) a Medical Research Council (MRC) Mental Health Data Pathfinder Award to King's College London; iii) an NIHR Senior Investigator Award.

Competing interests: RD declares previous research funding received from Janssen. RS has received research support in the last 5 years from Roche, Janssen, GSK and Takeda.

Ethics approval: Oxford C Research Ethics Committee

Data sharing statement: Data must remain within the SLaM firewall and any requests to access the data can be addressed to cris.administrator@kcl.ac.uk.

For peer review only

For peer review only - http://bmjopen.bmj.com/site/about/guidelines.xhtml

BMJ Open

Clinician- recalled Quoted Speech in Electronic Health Records and Risk of Suicide Attempt: A Case-Crossover Study

Journal:	*BMJ Open*
Manuscript ID	bmjopen-2019-036186.R1
Article Type:	Original research
Date Submitted by the Author:	18-Feb-2020
Complete List of Authors:	Jayasinghe, Lasantha; IoPPN Department of Psychological Medicine,<br>Bittar, André; IoPPN Department of Psychological Medicine<br>Dutta, Rina; Kings College London, Institute of Psychiatry, Psychology and Neuroscience; South London and Maudsley NHS Foundation Trust<br>Stewart, Robert; King's College London, Institute of Psychiatry; South London and Maudsley NHS Foundation Trust
**Primary Subject Heading**:	Mental health
Secondary Subject Heading:	Health informatics, Mental health
Keywords:	PSYCHIATRY, Suicide & self-harm < PSYCHIATRY, Health informatics < BIOTECHNOLOGY & BIOINFORMATICS

SCHOLARONE™
Manuscripts

I, the Submitting Author has the right to grant and does grant on behalf of all authors of the Work (as defined in the below author licence), an exclusive licence and/or a non-exclusive licence for contributions from authors who are: i) UK Crown employees; ii) where BMJ has agreed a CC-BY licence shall apply, and/or iii) in accordance with the terms applicable for US Federal Government officers or employees acting as part of their official duties; on a worldwide, perpetual, irrevocable, royalty-free basis to BMJ Publishing Group Ltd ("BMJ") its licensees and where the relevant Journal is co-owned by BMJ to the co-owners of the Journal, to publish the Work in this journal and any other BMJ products and to exploit all rights, as set out in our licence.

The Submitting Author accepts and understands that any supply made under these terms is made by BMJ to the Submitting Author unless you are acting as an employee on behalf of your employer or a postgraduate student of an affiliated institution which is paying any applicable article publishing charge ("APC") for Open Access articles. Where the Submitting Author wishes to make the Work available on an Open Access basis (and intends to pay the relevant APC), the terms of reuse of such Open Access shall be governed by a Creative Commons licence – details of these licences and which Creative Commons licence will apply to this Work are set out in our licence referred to above.

Other than as permitted in any relevant BMJ Author's Self Archiving Policies, I confirm this Work has not been accepted for publication elsewhere, is not being considered for publication elsewhere and does not duplicate material already published. I confirm all authors consent to publication of this Work and authorise the granting of this licence.

For peer review only

Clinician- recalled Quoted Speech in Electronic Health Records and Risk of Suicide Attempt: A Case-Crossover Study

Lasantha Jayasinghe[1], André Bittar[1], Rina Dutta[*,1,2], Robert Stewart[*,1,2]
[1] *Institute of Psychiatry, Psychology and Neuroscience, King's College London, London, UK,*
[2] *South London and Maudsley NHS Foundation Trust, London, UK*

[*]*contributed equally to this work*

Correspondence to: Lasantha Jayasinghe. lasantha.jayasinghe@kcl.ac.uk

Other Author emails:

André Bittar: andre.bittar@kcl.ac.uk
Rina Dutta: rina.dutta@kcl.ac.uk
Robert Stewart: robert.stewart@kcl.ac.uk

Keywords: electronic health records, suicide attempt, mental health, case crossover design, natural language processing

Word count: 3524

For peer review only - http://bmjopen.bmj.com/site/about/guidelines.xhtml

ABSTRACT

Objective: Clinician narrative style in electronic health records (EHRs) has rarely been investigated. Clinicians sometimes record brief quotations from patients, possibly more frequently when higher risk is perceived. We investigated whether the frequency of quoted phrases in an EHR was higher in time periods closer to a suicide attempt.

Design: A case-crossover study was conducted in a large mental health records database. A natural language processing tool was developed using regular expression matching to identify text occurring within quotation marks in the EHR.

Setting: Electronic records from a large mental healthcare provider serving a geographic catchment of 1.3 million residents in South London were linked with hospitalisation data.

Participants: 1,503 individuals were identified as having a hospitalised suicide attempt from 1st April 2006 to 31st March 2017 with at least one document in both the case period (1 to 30 days prior to admission) and the control period (61 to 90 days prior to admission).

Outcome measures: The number of quoted phrases in the control as compared to the case period.

Results: Both attended (OR 1.05, 95% CI 1.02 – 1.08) and non-attended (OR 1.15, 95% CI 1.04 – 1.26) clinical appointments were independently higher in the case compared to control period, while there was no difference in mental healthcare hospitalisation (OR 0.99, 95% CI 0.98 – 1.01). In addition, there was no difference in the levels of quoted text between the comparison time periods (OR 1.09, 95% CI 0.91 - 1.30).

Conclusions: This study successfully developed an algorithm to identify quoted speech in text fields from routine mental healthcare records. Contrary to the hypothesis, no association between this exposure and proximity to a suicide attempt was found; however, further evaluation is warranted on the way in which clinician-perceived risk might be feasibly characterised from clinical text.

For peer review only - http://bmjopen.bmj.com/site/about/guidelines.xhtml

ARTICLE SUMMARY

Strengths and limitations of this study

- A larger sample size (1,503 patients) than previous related studies.
- The case-crossover design eliminates between-patient differences as potential confounding factors, making the results more robust.
- Time-varying factors that could potentially cause confounding were included as covariates in the regression.
- 90% of the 14,960 patients making a suicide attempt in the case period could not be analysed, due to no documentation in the control period, although analysis appears representative.

For peer review only - http://bmjopen.bmj.com/site/about/guidelines.xhtml

INTRODUCTION

Around 800,000 deaths a year internationally are estimated to be the result of suicide (1). In the UK, 5,821 deaths were attributed to suicide in 2017 (2). Given that predictions of suicide risk have not improved significantly in the last 50 years, new data-driven methods would be welcomed, and a recent meta-analysis concluded a shift away from risk factors to risk algorithms (3,4).

Electronic Health Record (EHR) data provide important potential opportunities in this respect, and for patients receiving mental health services they hold a rich source of longitudinal data leading up to recorded suicide attempts. There are also benefits over traditional assessment methods such as questionnaires, in which respondents may not answer accurately due to perceived stigma (5–7) or limited recall (5). Commonly, predictive studies have sought to understand suicide risk in terms of the structured data within EHRs, such as diagnostic codes (8,9). However, recent studies have begun to examine the value of including unstructured EHR data for predicting suicide risk. For example, text-mined EHR data were found to produce more accurate models of suicidal behaviour in a sample of Gulf War veterans (10), and important information such as recorded suicidal ideation and past attempts have been successfully identified in mental healthcare text using natural language processing (11).

For peer review only

Previous studies using EHRs to investigate suicide risk have focussed on identifying individuals at risk of suicide from those not at risk (i.e. between-person variation). These have included Bayesian modelling to identify patients at risk of suicide attempt where patients had made 3 or more healthcare visits in a retrospective cohort (12), Cox regression to develop a 10-year probability prediction model for death by suicide in a sample from the Korean National Health Insurance Service (13), and neural networks applied to the EHRs of UK patients to identify those most at risk of suicide (14). Simon and colleagues (15) examined the records of US patients across seven health systems and determined that incorporating EHR data provided significant improvements to existing suicide

For peer review only - http://bmjopen.bmj.com/site/about/guidelines.xhtml

prediction methods, while Karmakar and others (16) developed a novel model using physical illness data from the EHR to predict suicide attempts. Walsh and others (9) used machine learning approaches to successfully identify patients who made a suicide attempt from those that self-harmed. Whilst this between-person investigation is an immensely important area of research, relatively little investigation has taken place using EHRs to investigate temporal changes associated with suicide risk (i.e. within-person associations), although it is known, for example, that risk is relatively high in relation to particular clinical events (e.g. shortly after discharge from inpatient care (17)), as well as showing seasonal fluctuation (18). The case-crossover design (19) was introduced as a method for studying transient effects on the risk of acute events. Previous research has employed this design to study suicidal behaviours in terms of negative life events (20), death of close relatives (21), substance abuse(22), and antidepressant drugs (23) (24). These studies gathered information from structured fields in registers, questionnaires and insurance records, but not directly from the free text in EHRs. Therefore, this study sought to determine transient changes within a patient's record over time that might contribute to the patient's risk of suicide.

Considering the source data for this study,the tendency has been to view the EHR as simply a factual summary of a patient's experience and symptoms. However, it is important to bear in mind that the EHR can also be viewed as a narrative account written from the perspective of the clinician and other healthcare professionals (25). Clinician reporting in the EHR varies widely and the nature/style of the reporting may provide additional information beyond what is actually recorded (26). A frequent comment in EHRs is a generic phrase such as 'No evidence of suicidal ideation', yet this raises the question of whether the clinician actually asked about suicide or just observed for signs indicating suicidality. A video analysis of out-patient visits in one study found that most questions about suicidal ideation were closed yes/no questions, and three-quarters of the questions were negatively phrased, inviting patients to confirm they were not feeling suicidal (27).

For peer review only - http://bmjopen.bmj.com/site/about/guidelines.xhtml

Trainee psychiatrists are often taught to use brief verbatim patient quotes to record a patient's actual words in order to provide better evidence for their decision making and also as part of medical defensive practice (28,29). In EHRs this may manifest in direct quotations of statements made by the patient, or by 'referencing' – where a clinician assigns the source of the text to someone other than themselves. Associations of recording style with perceived risk are supported by a higher relative frequency of third-person pronoun use found in groups of veterans who had died from suicide compared to a group of service users who were still alive (30). In that study the use of a group of words related to referencing did not vary significantly between the two groups, but the authors did not report on directly quoted speech in quotation marks. However, another investigation found that quoting 'he/she says' increased in records of clinician-patient interactions that involved communication of bad news between doctor and patient (31).

Clinical recording style has received little investigation in the field of suicidology. To our knowledge, research into the use of quotation marks to record verbatim patient statements in notes has not been conducted to date. We therefore sought to investigate the hypothesis that the frequency of quoted phrases in an EHR would be indicative of higher perceived immediate risk, in that they would be more frequent in time periods closer to a suicide attempt. Assumming that this reflects a transient change within a patient from one time period to another, a case-crossover design was used in this study.

For peer review only

For peer review only - http://bmjopen.bmj.com/site/about/guidelines.xhtml

METHODS

Study sample

The mental health records used in this study were de-identified copies of those from the South London and Maudsley (SLaM) NHS Foundation Trust, assembled using its Clinical Record Interactive Search (CRIS) platform, which currently accesses mental healthcare records for over 400,000 patients (32). CRIS, as a data resource for secondary analysis, has IRB approval from Oxford Research Ethics Committee C (reference 18/SC/0372). SLaM provides comprehensive, near-monopoly mental healthcare services to a geographic catchment of around 1.3m residents in four boroughs of south London, as well as some national specialist services. CRIS data have been linked to the Hospital Episode Statistics (HES) database, which contains details of all admissions, accident and emergency attendances and outpatient appointments at NHS hospitals in England (33). This CRIS-HES linkage was used to determine suicide-related hospital admissions for individuals between 1st April 2006 and 31st March 2017 inclusive, identifying 14,960 unique patients with at least one suicide attempt, indicated by the presence of any of the following ICD codes: X6*, X7*, X80-4*, Y1*, Y2*, Y30-4*, Y87*, associated with a hospitalisation lasting at least 24 hours (i.e. starting and ending on different dates). From these, a subset of 1,503 patients (10.0%) were identified who had at least one document from mental healthcare in both the case and control period (see below) and who thus provided sufficient data for the analysis. Of these 1503 patients, 877 (58.3%) have at least 1 quotation in the control period, 919 (61.1%) have at least 1 quotation in the case period, and 625 (41.6%) have at least 1 quotation in both the control and case periods. In addition to the text-derived data, demographics including age, gender and ethnicity were extracted to describe the cohort.

Patient involvement

For peer review only - http://bmjopen.bmj.com/site/about/guidelines.xhtml

We did not directly incorporate patient and public involvement (PPI) into this particular study but the SLaM BRC Case Register used in the analysis was developed with extensive PPI and is overseen by a committee that includes service-user representatives.

Case-crossover design

In this study the case-crossover design was used to compare the occurrence of quoted phrases in clinical text between the period just prior to a suicide attempt and a control period, within the same individualThe advantage of this design is that although comparisons cannot be made between individuals, individual confounders that do not vary over time, such as gender, race and genetics are eliminated (34). Additionally, this design is particularly well suited to EHR data research which allows for sufficiently large samples experiencing a given event, which is not possible in the traditional cohort study; also EHR data does not depend on recollection of past events, which may lead to recall biases. On the other hand the design requires data within appropriate comparison periods and may therefore need to be restricted to clinical subgroups where this is present; in addition, other time-varying factors may act as confounders and need to be captured and quantified. For the analyses presented here, the index date was the hospital admission date for a first suicide attempt. The case period was defined pragmatically and *a priori* as 1 to 30 days prior to the index date and the control period was set at 61 to 90 days prior to this date.

Time-variant factors

Considering other potential differences between the two comparison time periods, the following variables were considered as confounding factors and included as covariates in the regression: i) number of face-to-face outpatient appointments attended, ii) number of appointments made and not attended, iii) number of inpatient bed days in SLaM. These were all included as potential

For peer review only - http://bmjopen.bmj.com/site/about/guidelines.xhtml

For peer review only

confounders, as differences in these variables in the two time periods would result in differences in the number of documents and therefore the number of quotations present per patient that might have been caused by factors other than the period effect.

Identification of quoted speech

A natural language processing (NLP) tool was developed using regular expression matching to identify text occurring within quotation marks in the EHR. To avoid mistaking apostrophes used in contractions for the start of quoted phrase, a quote followed by a sequence (‘c’, ‘d’, ‘e’, ‘m’, ‘n’, ‘s’, ‘t’ , ‘ve’, ‘re’, ‘s’, ‘ll’, ‘all’) was treated as an apostrophe not a quote. A similar check was performed for end quotes. Once a quoted phrase was identified, any sub-quotations occurring within that quote were assumed to be part of the larger quotation. The length of quoted phrases was allowed to vary from one word to more than a paragraph; however, a maximum length of 1,500 characters was applied to avoid extracting the entire text where a quote was not properly closed. Phrases that consisted only of emails or URLs were removed using standard regular expression pattern matching and substitution procedures.

The performance of the algorithm was tested on random samples of both clinical correspondence and case note documents from CRIS (Table 1), with a precision of 0.98, recall of 0.95 and F-Score of 0.96 across the whole test sample. To test the performance in the model sample, 15 random documents identified by the algorithm as containing quotations and 10 without quotations were examined in both the case and control periods. In summary, 49 documents were evaluated, yielding a precision of 0.92, recall of 0.93, F-score of 0.92 and accuracy 0.86. One document was eliminated from the analysis due to incorrect syntax. The majority of inconsistences between the algorithm and annotated quotations were due to grammatical inconsistences such as omitting one of the pair of quotation marks or mismatching two different quotation mark styles. This was not amended in the regular expression matching pattern as it was felt to be more likely to produce false positives than genuine quotes. It is important to note that the algorithm was not designed to identify the speaker

For peer review only - http://bmjopen.bmj.com/site/about/guidelines.xhtml

of the quoted text; however, analysis of a random sample of 25 documents each from the control and case period showed that quotations referred to patient speech in 70.5% and 95.8% of instances respectively.

Statistical Analysis

Data were cleaned using standard Python (version 3.6.8) libraries. Statistical analyses (paired *t*-tests and conditional logistic regressions) were performed in R (version 3.6.0). Two-tailed paired *t*-tests were carried out to investigate differences in the number of quoted phrases between the control and case periods. Quotations per token were used to normalise for document length. Main analyses used conditional logistic regression to examine the association between the number of quoted phrases in the control and cases periods.

For peer review only
For peer review only - http://bmjopen.bmj.com/site/about/guidelines.xhtml

RESULTS

Cohort Characteristics

The demographics of the study cohort and the wider sample of patients with at least one suicide attempt in the time period of analysis, are described in Table 2. Of the 1,503 individuals in the cohort the majority were female, the mean age at first presentation to SLaM was 34.8 years (*sd* = 16.1 years), with 64.5% aged 40 or under. The majority were of White European background, and the next largest ethnic group was Black. These characteristics of the study cohort were broadly similar to those of the wider sample (Table 2). Individuals in the study cohort had approximately the same number of documents in the control period (*mean* = 14.7, *sd* = 28.5) and case period (*mean* = 15.3, *sd* = 28.9); *p* = 0.381.

Univariate Analyses

Univariate analyses of the individual covariates were carried out to investigate differences between the case and the control period (Table 3). The univariate distributions were non-normal, displaying high peaks and long tails; however, they were compared with paired *t*-tests, which are robust under this distribution (35). Face-to-face outpatient attendances and non-attendances were significantly higher in the case period compared to the control period, but there was no significant difference in the number of inpatient bed days. With regard to the exposure of interest, no difference was found in the number of quoted phrases for the patient group in the case period compared to the control period.

Conditional Logistic Regression

Univariate and multivariate conditional logistic regression results are presented in Table 4. Considering presence or not of quoted text as a binary variable, although an association of borderline significance was present in the unadjusted model (OR 1.17, 95% CI 0.99 - 1.38), this was attenuated substantially after adjustment for other covariates (OR 1.09, 95% CI 0.91 - 1.30) with the

For peer review only - http://bmjopen.bmj.com/site/about/guidelines.xhtml

majority of the attenuation occurring following adjustment for level of face-to-face contact. The full details of the coefficients in the quotations binary model, including face-to-face contacts, appointments not attended and inpatient bed days as covariates are presented in Table 5. Associations between number of quoted text instances and case vs. control periods were close to the null in all models, while both attended (OR 1.05, 95% CI 1.02 – 1.08) and non-attended (OR 1.15, 95% CI 1.04 – 1.26) appointments were independently higher in case compared to control periods.

For peer review only

For peer review only - http://bmjopen.bmj.com/site/about/guidelines.xhtml

DISCUSSION

To our knowledge, this was the first study using a case-crossover design to examine whether changes in the frequency of clinicians-recalled quoted text might serve as an indicator of suicide risk in patients. In summary, we found no association between the frequency of quoted phrases between periods close-to and less-close-to the occurrence of an attempted suicide event, although there were differences in other metrics between the time periods, notably increased numbers of both attended and non-attended appointments closer to the event in question.

Previous research has been sparse on mental health risk implications of different reporting styles in clinical records. As mentioned, one previous study had reported no association between referencing text as a construct and suicidality (30); however, we felt that the association of text in quotation marks was worth evaluating as an alternative marker, not only because of the potential strengths of a case-crossover design, but also because of another study's findings that quoted text used in particular clinical circumstances is associated with higher perceived risk (31). One possible difference between that study and ours is that we used quotation marks to identify quoted phrases, rather than attempting to identify the speaker, or exchanges of views between clinician and patient. Thus, extending the definition of quoted text to the identification of relevant pronouns would be a worthwhile avenue for future research.

Additional findings of our study were that increased face-to-face contact and failure to attend appointments were significantly more common in the case compared to control period and thus were markers of higher risk status. These are clinically plausible associations and support the robustness of the case crossover design for this outcome. Patients with increased face-to-face contact in the case period would presumably have a greater number of documents and potentially quotations than in the control period, whilst this would be less likely in those failing to attend appointments in the case period; supporting this, adjusting the association of interest for face-to-

For peer review only - http://bmjopen.bmj.com/site/about/guidelines.xhtml

For peer review only

face contacts resulted in substantial attenuation, whereas adjusting for non-attendances made little difference to coefficients.

The data for this study were drawn from a much larger source than the previous studies: for example, a narrative analysis of 4 medical interviews (31) or a sample of 63 veterans with the equivalent number of controls (30). Another key strength of this study is that between-patient confounding factors are eliminated through the case-crossover design, which renders a more robust analysis than that attempted previously, and the positive associations with attended and non-attended appointments supports the robustness of the time period comparison. As well as difference in individual-level characteristics, the design should also have equalised most clinical/service related potential confounders, such as workflow, providers and system-level factors, particularly as the comparison time periods are relatively proximal to each other and are unlikely to contain substantial differences in service type. However, it is important to bear in mind that the case-crossover design only addresses within-individual variations as a risk factor, and clearly cannot be used to test associations with characteristics that vary between individuals.

A limitation of our study is that we were only able to analyse a small sample of the 14,960 patients who had made a suicide attempt during the time period of interest, 79% of whom had no prior contact with SLaM (i.e. no documents at all in the case and control periods), although those analysed appeared representative of the source sample on metrics investigated. Consequently, the findings are limited to patients who were already seeking mental health treatment. Previous research shows that in a geographically diverse study of people dying by suicide in the US, in the month prior to death, only 5% of cases received psychiatric treatment, but over 60% had made primary and secondary care visits in the preceding year (36). Potential linkage of information beyond mental healthcare to primary and acute care might prove useful for predictive modelling, not only in the

For peer review only - http://bmjopen.bmj.com/site/about/guidelines.xhtml

mining of text but also through a wider variety of structured information on the nature and level of service contact.

The absent association of interest might possibly have arisen because the use of quotations is a relatively invariant factor based on individual clinician linguistic style; it was not possible to account for this limitation in the analysis and this is important as different individuals may have differing tendencies to quote patient speech. Another limitation was that we were not able to determine the distance between the recording of speech as it is produced and when the transfer electronically occurred. It is common practice for clinicians to hand write notes and transfer them to electronic records later, so we cannot precisely determine if quotations were actually produced in the case or control periods, and this may have affected the outcomes. Additionally, this algorithm did not seek to identify the speaker of the quoted text, although the majority of cases in random evaluated samples did represent the patient's speech. Identification of the speaker would clearly prove an interesting avenue for future research.

Despite limitations, this method is a novel approach, as identifying quotations in clinical text has not been a focal point of research to date. We believe that this is a potentially fruitful avenue of investigation, as quotations may have referenced a clinician variable that has not been previously investigated. Furthermore, as indicated, quoted text was relatively common for the subset who had documentation in the comparison periods of interest. Although findings for the primary hypothesis were null in this comparison, the development of an NLP algorithm has provided the means for the creation of a database of quotations across CRIS, generating a much larger sample that might of interest for future work: both analysing the occurrence or not of quoted text, and potentially the content of such quotations for further characterisation (e.g. speech patterns, sentiment).

For peer review only - http://bmjopen.bmj.com/site/about/guidelines.xhtml

In conclusion, this study found that there was no difference in the levels of quoted text for individuals at 1 to 30 days versus 61 to 90 days prior to a suicide attempt. However, the successful identification of quoted speech within mental healthcare EHRs may have other applications, and there may be fruitful progress to be made in automating the extraction of such text and analysing what the clinician thinks it is important to emphasise from the patient's account.

For peer review only

For peer review only - http://bmjopen.bmj.com/site/about/guidelines.xhtml

REFERENCES

1. WHO: WHO | National suicide prevention strategies: progress, examples and indicators. WHO , 2019.Available from: https://www.who.int/mental_health/suicide-prevention/national_strategies_2019/en/

2. ONS: Suicides in the UK: 2017 registrations [Internet]. Off. Natl. Stat. , 2018.Available from: https://www.ons.gov.uk/peoplepopulationandcommunity/birthsdeathsandmarriages/deaths/bulletins/suicidesintheunitedkingdom/2017registrations

3. Jaroszewski MC, Chang AC, Nock BP: Risk Factors for Suicidal Thoughts and Behaviors: A Meta-Analysis of 50 Years of Research. Psychological Bulletin , 2016.Available from: http://dx.doi.org/10.1037/bul0000084CITATIONAdvanceonlinepublication.http://dx.doi.org/10.1037/bul0000084

4. Velupillai S, Hadlaczky G, Baca-Garcia E, et al.: Risk Assessment Tools and Data-Driven Approaches for Predicting and Preventing Suicidal Behavior. Frontiers in psychiatry 10: 36, 2019.Available from: http://www.ncbi.nlm.nih.gov/pubmed/30814958

5. Denneson LM, Basham C, Dickinson KC, et al.: Suicide Risk Assessment and Content of VA Health Care Contacts Before Suicide Completion by Veterans in Oregon. Psychiatric Services 61: 1192–1197, 2010.Available from: http://psychiatryonline.org/doi/abs/10.1176/ps.2010.61.12.1192

6. Bowers L, Banda T, Nijman H: Suicide Inside. The Journal of Nervous and Mental Disease 198: 315–328, 2010.Available from: https://insights.ovid.com/crossref?an=00005053-201005000-00001

7. Schulberg HC, Bruce ML, Lee PW, et al.: Preventing suicide in primary care patients: the primary care physician's role. General Hospital Psychiatry 26: 337–345, 2004.Available from: https://www.sciencedirect.com/science/article/pii/S016383430400088X

8. Tran T, Luo W, Phung D, et al.: Risk stratification using data from electronic medical records better predicts suicide risks than clinician assessments. BMC Psychiatry 14: 76, 2014.Available

For peer review only - http://bmjopen.bmj.com/site/about/guidelines.xhtml

from: http://bmcpsychiatry.biomedcentral.com/articles/10.1186/1471-244X-14-76

9. Walsh CG, Ribeiro JD, Franklin JC: Predicting risk of suicide attempts over time through machine learning. Clinical Psychological Science 5: 457–469, 2017.

10. Ben-Ari A, Hammond K: Text Mining the EMR for Modeling and Predicting Suicidal Behavior among US Veterans of the 1991 Persian Gulf War [Internet]; in 2015 48th Hawaii International Conference on System Sciences. IEEE, 2015 [cited 2019 Jun 19]. Available from: http://ieeexplore.ieee.org/document/7070197/

11. Fernandes AC, Dutta R, Velupillai S, et al.: Identifying Suicide Ideation and Suicidal Attempts in a Psychiatric Clinical Research Database using Natural Language Processing. Scientific Reports 8: 7426, 2018.Available from: http://www.nature.com/articles/s41598-018-25773-2

12. Barak-Corren Y, Castro VM, Javitt S, et al.: Predicting Suicidal Behavior From Longitudinal Electronic Health Records. American Journal of Psychiatry 174: 154–162, 2017.Available from: http://ajp.psychiatryonline.org/doi/10.1176/appi.ajp.2016.16010077

13. Choi SB, Lee W, Yoon JH, et al.: Ten-year prediction of suicide death using Cox regression and machine learning in a nationwide retrospective cohort study in South Korea. Journal of Affective Disorders 231: 8–14, 2018.

14. DelPozo-Banos M, John A, Petkov N, et al.: Using Neural Networks with Routine Health Records to Identify Suicide Risk: Feasibility Study. JMIR mental health 5: e10144, 2018.Available from: http://www.ncbi.nlm.nih.gov/pubmed/29934287

15. Simon GE, Johnson E, Lawrence JM, et al.: Predicting Suicide Attempts and Suicide Deaths Following Outpatient Visits Using Electronic Health Records. American Journal of Psychiatry 175: 951–960, 2018.Available from: http://ajp.psychiatryonline.org/doi/10.1176/appi.ajp.2018.17101167

16. Karmakar C, Luo W, Tran T, et al.: Predicting Risk of Suicide Attempt Using History of Physical Illnesses From Electronic Medical Records. JMIR mental health 3: e19, 2016.Available from: http://www.ncbi.nlm.nih.gov/pubmed/27400764

For peer review only

For peer review only - http://bmjopen.bmj.com/site/about/guidelines.xhtml

17. Forte A, Buscajoni A, Fiorillo A, et al.: Suicidal Risk Following Hospital Discharge: A Review. Harv. Rev. Psychiatry. 27: 209–216, 2019.

18. Woo J-M, Okusaga O, Postolache TT: Seasonality of Suicidal Behavior. International Journal of Environmental Research and Public Health 9: 531–547, 2012.Available from: http://www.mdpi.com/1660-4601/9/2/531

19. Maclure M: The Case-Crossover Design: A Method for Studying Transient Effects on the Risk of Acute Events. American Journal of Epidemiology 133: 144–153, 1991.Available from: https://doi.org/10.1093/oxfordjournals.aje.a115853

20. Liu BP, Zhang J, Chu J, et al.: Negative life events as triggers on suicide attempt in rural China: a case-crossover study. Psychiatry Research 276: 100–106, 2019.

21. Mogensen H, Möller J, Hultin H, et al.: Death of a Close Relative and the Risk of Suicide in Sweden—A Large Scale Register-Based Case-Crossover Study. PLOS ONE 11: e0164274, 2016.Available from: https://dx.plos.org/10.1371/journal.pone.0164274

22. Bagge CL, Borges G: Acute Substance Use as a Warning Sign for Suicide Attempts. The Journal of Clinical Psychiatry 78: 691–696, 2017.Available from: http://www.psychiatrist.com/jcp/article/pages/2017/v78n06/v78n0610.aspx

23. Björkenstam C, Möller J, Ringbäck G, et al.: An Association between Initiation of Selective Serotonin Reuptake Inhibitors and Suicide - A Nationwide Register-Based Case-Crossover Study. PLoS ONE 8: e73973, 2013.Available from: http://dx.plos.org/10.1371/journal.pone.0073973

24. Sung HG, Li J, Nam JH, et al.: Concurrent use of benzodiazepines, antidepressants, and opioid analgesics with zolpidem and risk for suicide: a case–control and case–crossover study. Social Psychiatry and Psychiatric Epidemiology 54: 1535–1544, 2019.

25. Aaslestad P: The patient as text: the role of the narrator in psychiatric notes, 1890-1990. CRC Press, 2016

26. Hunter KM, Montgomery K: Doctors' stories: The narrative structure of medical knowledge.

For peer review only - http://bmjopen.bmj.com/site/about/guidelines.xhtml

Princeton University Press, 1993

27. McCabe R, Sterno I, Priebe S, et al.: How do healthcare professionals interview patients to assess suicide risk? BMC Psychiatry 17: 122, 2017.Available from: http://bmcpsychiatry.biomedcentral.com/articles/10.1186/s12888-017-1212-7

28. Assessing and managing risk in mental health services - GOV.UK [Internet]Available from: https://www.gov.uk/government/publications/assessing-and-managing-risk-in-mental-health-services

29. Roth, Lorraine S: Writing progress notes: 10 dos and don'ts [Internet]. MDedge Psychiatry. 4(2):63-66, 2005.Available from: https://www.mdedge.com/psychiatry/article/59861/writing-progress-notes-10-dos-and-donts

30. Leonard Westgate C, Shiner B, Thompson P, et al.: Evaluation of Veterans' Suicide Risk With the Use of Linguistic Detection Methods. Psychiatric Services 66: 1051–1056, 2015.Available from: http://psychiatryonline.org/doi/10.1176/appi.ps.201400283

31. Van DeMieroop D: The quotative 'he/she says' in interpreted doctor–patient interaction. Interpreting 14: 92–117, 2012.Available from: http://www.jbe-platform.com/content/journals/10.1075/intp.14.1.05mie

32. Perera G, Broadbent M, Callard F, et al.: Cohort profile of the South London and Maudsley NHS Foundation Trust Biomedical Research Centre (SLaM BRC) Case Register: current status and recent enhancement of an Electronic Mental Health Record-derived data resource. BMJ open 6: e008721, 2016.Available from: http://www.ncbi.nlm.nih.gov/pubmed/26932138

33. Hospital Episode Statistics (HES) - NHS Digital [Internet]Available from: https://digital.nhs.uk/data-and-information/data-tools-and-services/data-services/hospital-episode-statistics

34. Mostofsky E, Coull BA, Mittleman MA: Analysis of Observational Self-matched Data to Examine Acute Triggers of Outcome Events with Abrupt Onset. Epidemiology 29,

For peer review only - http://bmjopen.bmj.com/site/about/guidelines.xhtml

2018.Available from: https://journals.lww.com/epidem/Fulltext/2018/11000/Analysis_of_Observational_Self_matched_Data_to.8.aspx

35. Peacock JL, Kerry SM, Balise RR: Presenting medical statistics from proposal to publication. Oxford University Press, 2017

36. Ahmedani BK, Simon GE, Stewart C, et al.: Health Care Contacts in the Year Before Suicide Death. Journal of General Internal Medicine 29: 870–877, 2014.Available from: http://link.springer.com/10.1007/s11606-014-2767-3

For peer review only

For peer review only - http://bmjopen.bmj.com/site/about/guidelines.xhtml

TABLES AND FIGURES

Table 1. Test set performance metrics for random documents selected to contain the quotation mark characters stated.

Minimum document length set to 500 characters, unless otherwise stated.

Quotation mark characters contained	Number of documents	Number of quotations	Precision	Recall	F-Score	Accuracy
<u>**Clinical correspondence**</u>						
“	10	111	0.96	0.95	0.95	0.91
”	10	50	0.98	0.98	0.98	0.96
"	9*	35	1.00	1.00	1.00	1.00
None of: “, ”, "	10	4	1.00	1.00	1.00	1.00
<u>**Case notes**</u>						
“	10	59	0.98	0.92	0.95	0.90

For peer review only - http://bmjopen.bmj.com/site/about/guidelines.xhtml

”	10	53	0.98	0.92	0.95	0.91
"	10	18	1.00	1.00	1.00	1.00
None of: “,”, "	10	0	-	-	-	-
At least 1 of: ‘,',“,”, "	10	10	0.91	1.00	0.95	0.91
All of: ‘,’, “,”, "	10	91	0.99	0.98	0.98	0.97
All of: ‘,’, “,”, " and document length > 1000 characters	30	264	0.97	0.92	0.95	0.90
Total	129	695	0.98	0.95	0.96	0.92

* One document removed as it was textually corrupt.

For peer review only

For peer review only - http://bmjopen.bmj.com/site/about/guidelines.xhtml

For peer review only

For peer review only - http://bmjopen.bmj.com/site/about/guidelines.xhtml

Table 2. **Demographic characteristics of the study sample (*n* = 1503) in comparison to all patients with hospitalised suicide attempt (N= 14,960)**

	Study sample		All suicide admissions	
Demographic Variables	***N***	***% Total***	***N***	***% Total***
**Gender**				
Female	932	62.0%	8463	56.6%
Male	571	38.0%	6488	43.4%
Unknown	0	0.0%	9	0.1%
**Ethnicity**				
White European	1083	72.1%	9805	65.5%
Black	274	18.2%	1512	10.1%
Asian	66	4.4%	615	4.1%
Other	66	4.4%	756	5.1%
Unknown	14	0.9%	2272	15.2%
**Age at index hospitalisation (y)**				
< 16	184	12.2%	1612	10.8%
16-20	205	13.6%	2204	14.7%

For peer review only - http://bmjopen.bmj.com/site/about/guidelines.xhtml

21-30	271	18.0%	3567	23.8%
31-40	310	20.6%	2935	19.6%
41-50	280	18.6%	2615	17.5%
51-60	156	10.4%	1195	8.0%
61+	97	6.5%	829	5.5%
Unknown	0	0.0%	3	0.0%

For peer review only

Table 3. Univariate Analyses - Paired *t*-test results

	Control period		Case period				
**Variable**	**mean**	**sd**	**mean**	**sd**	**Difference**	**95% CI**	***p*-value**
Face-to-face contact	2.47	3.71	2.92	4.14	0.441	0.225, 0.657	<0.001
Appointment not attended	0.38	1.00	0.47	1.13	0.098	0.036, 0.161	0.002
Inpatient bed days	2.29	7.13	2.05	6.69	-0.242	-0.620, 0.134	0.207
Quotations per token*	0.16	0.25	0.16	0.23	-0.0004	-0.0158, 0.0149	0.956

* Tokens refer to word tokens as determined by the python nltk Regex Word Tokenizer

For peer review only - http://bmjopen.bmj.com/site/about/guidelines.xhtml

Table 4. Conditional logistic regression models for the association between levels of quoted speech and time period prior to hospitalised suicide attempt

Characteristic	Odds Ratio	95% CI	*p*-value
Total sample (n = 1503)			
**quotations_binary**			
Unadjusted	1.17	0.99, 1.38	0.073
Adjusted for face-to-face contacts	1.08	0.90, 1.28	0.411
Adjusted for dna	1.16	0.98, 1.37	0.087
Adjusted for no. of inpatient bed days	1.18	1.00, 1.40	0.050
Adjusted for all covariates	1.09	0.91, 1.30	0.346
**quotations_per_token***			
Unadjusted	0.99	0.71, 1.38	0.956
Adjusted for face-to-face contacts	0.93	0.67, 1.31	0.693
Adjusted for dna	0.99	0.71, 1.39	0.971

For peer review only - http://bmjopen.bmj.com/site/about/guidelines.xhtml

For peer review only

Adjusted for no. of inpatient bed days	0.99	0.71, 1.39	0.969
Adjusted for all covariates	0.94	0.67, 1.32	0.735

* Tokens refer to word tokens as determined by the python nltk Regex Word Tokenizer

For peer review only

For peer review only - http://bmjopen.bmj.com/site/about/guidelines.xhtml

Table 5. Full conditional logistic regression model for covariates associated with time period prior to hospitalised suicide attempt

Characteristic	Odds Ratio	95% CI	*p*-value
Total sample (n = 1503)			
Quotations (binary)	1.09	0.91, 1.30	0.346
Face-to-face contacts	1.05	1.02, 1.08	0.001
Appointments not attended	1.15	1.04, 1.26	0.004
Inpatient bed days	0.99	0.98, 1.01	0.211

For peer review only

For peer review only - http://bmjopen.bmj.com/site/about/guidelines.xhtml

AUTHOR STATEMENT

Contributors: RD and RS conceived the study design. LJ wrote the paper and analysed the data. LJ also led the development of the quoted speech algorithm with input from AB. RD and RS provided clinical insight on the paper and supervisory guidance. All authors provided critical input for the paper and approved the submission.

Funding: LJ and RD are part-funded by the NIHR Specialist Biomedical Research Centre for Mental Health at the South London and Maudsley NHS Foundation Trust and Institute of Psychiatry, King's College London. RD is also funded by a Clinician Scientist Fellowship (project e-HOST-IT) from the Health Foundation in partnership with the Academy of Medical Sciences, which also funds AB. RS is part-funded by: i) the NIHR Specialist Biomedical Research Centre for Mental Health at the South London and Maudsley NHS Foundation Trust and Institute of Psychiatry, King's College London; ii) a Medical Research Council (MRC) Mental Health Data Pathfinder Award to King's College London; iii) an NIHR Senior Investigator Award.

Competing interests: RD declares previous research funding received from Janssen. RS has received research support in the last 5 years from Roche, Janssen, GSK and Takeda.

Ethics approval: Oxford C Research Ethics Committee

Data sharing statement: Data must remain within the SLaM firewall and any requests to access the data can be addressed to cris.administrator@kcl.ac.uk.

For peer review only

For peer review only - http://bmjopen.bmj.com/site/about/guidelines.xhtml

The RECORD statement – checklist of items, extended from the STROBE statement, that should be reported in observational studies using routinely collected health data.

	Item No.	STROBE items	Location in manuscript where items are reported	RECORD items	Location in manuscript where items are reported
**Title and abstract**					
	1	(a) Indicate the study's design with a commonly used term in the title or the abstract (b) Provide in the abstract an informative and balanced summary of what was done and what was found		RECORD 1.1: The type of data used should be specified in the title or abstract. When possible, the name of the databases used should be included.<br><br>RECORD 1.2: If applicable, the geographic region and timeframe within which the study took place should be reported in the title or abstract.<br><br>RECORD 1.3: If linkage between databases was conducted for the study, this should be clearly stated in the title or abstract.	Title and abstract<br><br>Abstract<br><br>Abstract
**Introduction**					
Background rationale	2	Explain the scientific background and rationale for the investigation being reported			Introduction paragraphs 1 - 6
Objectives	3	State specific objectives, including any prespecified hypotheses			Introduction Paragraph 7
**Methods**					
Study Design	4	Present key elements of study design early in the paper			Methods: study sample, case-crossover design & time-variant fctors sections

For peer review only

Setting	5	Describe the setting, locations, and relevant dates, including periods of recruitment, exposure, follow-up, and data collection			Methods: study sample section
Participants	6	*(a) Cohort study* - Give the eligibility criteria, and the sources and methods of selection of participants. Describe methods of follow-up<br>*Case-control study* - Give the eligibility criteria, and the sources and methods of case ascertainment and control selection. Give the rationale for the choice of cases and controls<br>*Cross-sectional study* - Give the eligibility criteria, and the sources and methods of selection of participants<br><br>*(b) Cohort study* - For matched studies, give matching criteria and number of exposed and unexposed<br>*Case-control study* - For matched studies, give matching criteria and the number of controls per case		RECORD 6.1: The methods of study population selection (such as codes or algorithms used to identify subjects) should be listed in detail. If this is not possible, an explanation should be provided.<br><br>RECORD 6.2: Any validation studies of the codes or algorithms used to select the population should be referenced. If validation was conducted for this study and not published elsewhere, detailed methods and results should be provided.<br><br>RECORD 6.3: If the study involved linkage of databases, consider use of a flow diagram or other graphical display to demonstrate the data linkage process, including the number of individuals with linked data at each stage.	Case-crossover study (individuals act as own controls)<br>Methods: study sample section<br><br>N/A<br><br>N/A
Variables	7	Clearly define all outcomes, exposures, predictors, potential confounders, and effect modifiers. Give diagnostic criteria, if applicable.		RECORD 7.1: A complete list of codes and algorithms used to classify exposures, outcomes, confounders, and effect modifiers should be provided. If these cannot be reported, an explanation should be provided.	Methods: study sample section<br>Methods: Identification of quoted speech
Data sources/ measurement	8	For each variable of interest, give sources of data and details of methods of assessment (measurement).			

For peer review only

		Describe comparability of assessment methods if there is more than one group			
Bias	9	Describe any efforts to address potential sources of bias			N/A
Study size	10	Explain how the study size was arrived at			Methods: study sample section
Quantitative variables	11	Explain how quantitative variables were handled in the analyses. If applicable, describe which groupings were chosen, and why			Methods
Statistical methods	12	(a) Describe all statistical methods, including those used to control for confounding<br>(b) Describe any methods used to examine subgroups and interactions<br>(c) Explain how missing data were addressed<br>(d) *Cohort study* - If applicable, explain how loss to follow-up was addressed<br>*Case-control study* - If applicable, explain how matching of cases and controls was addressed<br>*Cross-sectional study* - If applicable, describe analytical methods taking account of sampling strategy<br>(e) Describe any sensitivity analyses			a) Methods, statistical analysis, case-crossover design and time-invariant factors sections.<br>b) N/A<br>c) Methods, study sample<br>d) N/A<br>e) N/A
Data access and cleaning methods		..		RECORD 12.1: Authors should describe the extent to which the investigators had access to the database	Methods: study sample section

For peer review only

				population used to create the study population.<br><br>RECORD 12.2: Authors should provide information on the data cleaning methods used in the study.	 <br>Methods: Identification of quoted speech section
Linkage		..		RECORD 12.3: State whether the study included person-level, institutional-level, or other data linkage across two or more databases. The methods of linkage and methods of linkage quality evaluation should be provided.	Methods: study sample section
**Results**					
Participants	13	(a) Report the numbers of individuals at each stage of the study (*e.g.*, numbers potentially eligible, examined for eligibility, confirmed eligible, included in the study, completing follow-up, and analysed)<br>(b) Give reasons for non-participation at each stage.<br>(c) Consider use of a flow diagram		RECORD 13.1: Describe in detail the selection of the persons included in the study (*i.e.,* study population selection) including filtering based on data quality, data availability and linkage. The selection of included persons can be described in the text and/or by means of the study flow diagram.	Methods: study sample section
Descriptive data	14	(a) Give characteristics of study participants (*e.g.*, demographic, clinical, social) and information on exposures and potential confounders<br>(b) Indicate the number of participants with missing data for each variable of interest<br>(c) *Cohort study* - summarise follow-up time (*e.g.*, average and total amount)			Results: Cohort Characteristics section.<br><br>Table 2.

For peer review only

Outcome data	15	*Cohort study* - Report numbers of outcome events or summary measures over time<br>*Case-control study* - Report numbers in each exposure category, or summary measures of exposure<br>*Cross-sectional study* - Report numbers of outcome events or summary measures			Table 3.
Main results	16	(a) Give unadjusted estimates and, if applicable, confounder-adjusted estimates and their precision (e.g., 95% confidence interval). Make clear which confounders were adjusted for and why they were included<br>(b) Report category boundaries when continuous variables were categorized<br>(c) If relevant, consider translating estimates of relative risk into absolute risk for a meaningful time period			Table 4.
Other analyses	17	Report other analyses done—e.g., analyses of subgroups and interactions, and sensitivity analyses			Table 5.
**Discussion**					
Key results	18	Summarise key results with reference to study objectives			Discussion, paragraph 1, 8
Limitations	19	Discuss limitations of the study, taking into account sources of potential bias or imprecision. Discuss both direction and magnitude of any potential bias		RECORD 19.1: Discuss the implications of using data that were not created or collected to answer the specific research question(s). Include discussion of misclassification bias, unmeasured confounding, missing data, and changing eligibility over	Discussion, paragraph 4 - 6

For peer review only

				time, as they pertain to the study being reported.	
Interpretation	20	Give a cautious overall interpretation of results considering objectives, limitations, multiplicity of analyses, results from similar studies, and other relevant evidence			Discussion, paragraph 2 -4
Generalisability	21	Discuss the generalisability (external validity) of the study results			Discussion, paragraph 2 -4
**Other Information**					
Funding	22	Give the source of funding and the role of the funders for the present study and, if applicable, for the original study on which the present article is based			Funding statement
Accessibility of protocol, raw data, and programming code		..		RECORD 22.1: Authors should provide information on how to access any supplemental information such as the study protocol, raw data, or programming code.	Data sharing statement

*Reference: Benchimol EI, Smeeth L, Guttmann A, Harron K, Moher D, Petersen I, Sørensen HT, von Elm E, Langan SM, the RECORD Working Committee. The REporting of studies Conducted using Observational Routinely-collected health Data (RECORD) Statement. *PLoS Medicine* 2015; in press.

*Checklist is protected under Creative Commons Attribution (CC BY) license.
